# Supplementary material for: Comparisons of Anatomical Characteristics and Transcriptomic Differences between Heterografts and Homografts in Pyrus L
Source: Plants (Basel). 2022 Feb 22;11(5):580. doi: 10.3390/plants11050580 (PMC8912356; doi:10.3390/plants11050580)
Supplement: Supplementary file 1 [file plants-11-00580-s001.zip › Supplementary Figure.pdf]

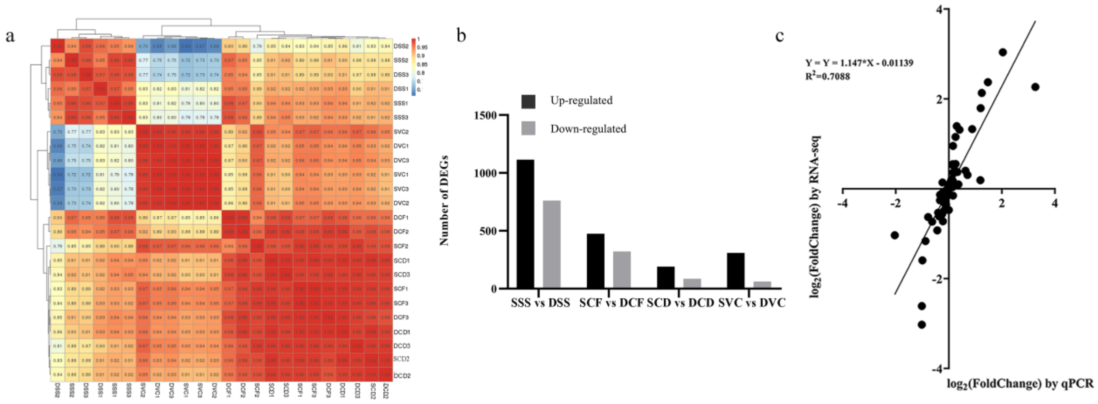

Fig S1. RNA-seq data and DEGs in different stages of homografts and heterografts. (a) Hierarchical clustering of 24 samples based on the correlation coeffificient (r^2) between each sample. SSS, SCF, SCD and SVC are different stages of homografts; DSS, DCF, DCD and DVC are different stages of heterografts. The color panel represents the r2 values. (b) Statistics of up- and downregulated DEGs for each pairwise comparison. Up- and downregulated DEGs are displayed in dark gray and light gray, respectively. (c) Correlation of expression changes observed by RNA-seq (Y-axis) and qPCR (X-axis).
