# Supplementary material for: Comparisons of Anatomical Characteristics and Transcriptomic Differences between Heterografts and Homografts in Pyrus L
Source: Plants (Basel). 2022 Feb 22;11(5):580. doi: 10.3390/plants11050580 (PMC8912356; doi:10.3390/plants11050580)
Supplement: Supplementary file 1 [file plants-11-00580-s001.zip › Supplementary Table.pdf]

Table S1. Oligonucleotide primers used in this study.

Genes	Forward primers (5'-3')	Reverse primers (5'-3')
Pbr003641.1	GTTACTCCACCTCCGTCTGC	AAGTCCGCAAACTGGCACTA
Pbr015955.1	GGGTTGCCACCTTTCGTACT	TTCAGAGGGATTTTCTGGGGC
Pbr016210.1	GTCCCCGGCGGAATCTC	ACATTCCCCACTCCCTCTCA
Pbr037054.1	AACGTGGCACTACCACTCAC	ACACGACGATGACACCCTTC
Pbr004636.1	TCGCAGGCGACCGTG	ACCAGACCCGAACAAACAGC
Pbr012591.1	AGCCCTGGAAAGAGTTCGTG	ACTTGTGCCTCCTGTTCGAG
Pbr021785.1	AGCTCCAGTGATGGCGAATC	ACCGATCCTATGGTCATGCG
Pbr035679.1	ATAGTTTTGGGAAACAGACCCTCA	CCACAGATGTTGGGGTGGAA
Pbr035767.1	ACGTGTGGGAGAGCAGATTG	TTCGCCAGCATGTCTTAGCA
Pbr039935.1	CCCAACAAACACTCTGCTGC	CGGGAAACCGTGGTAATCCA
Pbr016153.1	ATGCTGAAAGGTCAGCTCCC	GTGAAGGATTCTTGGCCCGA
Pbr010535.1	CTATGTTCCCATCTGGCGGG	CCAGATCGGCCTTCTTTGGT
Pbr015152.1	CGGGATTATCATCGACGGCA	TGACAGCATTTGCTGCACAC
Pbr015699.1	CGGGTTTGGCTAGGGACATT	TCTCTGTTTTCACTCTCATTTTCCA
Pbr036247.1	CGGCCGAGTCAAACACAATC	GGGTTGGTGATCACATCGGA
Bglu13-Pbr042200.2	TAACAGCATGTGCATGAGCG	ATGCATCAGGAGGATTCGCA
ACTIN (Internal control)	CGAAATCGGTAGACGCTACG	GGGGATAGAGGGACTTGAAC

Table S2. Offline statistics of every samples.

Sample	Raw Data(bp)	Clean Data(bp)	Q30(bp)	Total Mapped	Uniquely Mapped
SSS1	6333136800	5818016400 (91.86%)	5972909388 (94.31%)	77.15	89.86
SSS2	6608074800	6092076000 (92.19%)	6252118229 (94.61%)	76.9	89.75
SSS3	6161696100	5679644700 (92.17%)	5813523993 (94.34%)	76.76	89.9
SCF1	6204202800	5712485100 (92.07%)	5856683482 (94.39%)	76.28	89.73
SCF2	6502094700	5981463300 (91.99%)	6149789384 (94.58%)	75.81	89.65
SCF3	6903761700	6374078400 (92.32%)	6529935843 (94.58%)	76.06	89.67
SCD1	7665027000	7073992800 (92.28%)	7234523101 (94.38%)	76.2	89.67
SCD1	7489773600	6917608500 (92.36%)	7079130068 (94.51%)	76.02	89.6
SCD1	6766602600	6211785600 (91.8%)	6414492576 (94.79%)	76.19	89.62
SVC1	6918927000	6369230100 (92.05%)	6565599591 (94.89%)	74.31	88.22

SVC2	6953560500	6414109800 (92.24%)	6577835964 (94.59%)	75.59	89.35
SVC3	6817350300	6283494900 (92.16%)	6423936417 (94.22%)	75.96	89.55
DSS1	6417945600	5864499600 (91.37%)	6087741142 (94.85%)	76.85	89.57
DSS2	6417737100	5922322200 (92.28%)	6076078145 (94.67%)	77.37	89.86
DSS3	6364395000	5875472700 (92.31%)	6033249407 (94.79%)	76.94	89.69
DCF1	6794866500	6249458100 (91.97%)	6430556239 (94.63%)	76.15	89.65
DCF2	6626759700	6104902800 (92.12%)	6248416039 (94.29%)	76.42	89.72
DCF3	6826981200	6273411900 (91.89%)	6457024022 (94.58%)	76.12	89.65
DCD1	7068651000	6531854400 (92.4%)	6669972250 (94.35%)	76.24	89.65
DCD2	6190027500	5718126300 (92.37%)	5862400313 (94.7%)	76.62	89.78
DCD3	5866962600	5394012000 (91.93%)	5559523244 (94.75)	76.26	89.72
DVC1	6171285600	5701008600 (92.37%)	5840311287 (94.63%)	75.67	89.52
DVC2	7225668600	6653327100 (92.07%)	6844742534 (94.72%)	76.09	89.4
DVC3	7669818000	7072310400 (92.2%)	7242565580 (94.42%)	75.75	89.39
